# Thalamocortical bistable switch as a theoretical model of fibromyalgia pathogenesis inferred from a literature survey

**DOI:** 10.1007/s10827-022-00826-8

**Published:** 2022-07-11

**Authors:** Ilaria Demori, Giulia Giordano, Viviana Mucci, Serena Losacco, Lucio Marinelli, Paolo Massobrio, Franco Blanchini, Bruno Burlando

**Affiliations:** 1 Department of Earth, Environmental and Life Sciences (DISTAV), University of Genova, Corso Europa, 26, 16132 Genoa, Italy; 2 Department of Industrial Engineering, University of Trento, via Sommarive, 9, 38123 Trento, Italy; 3 School of Science, Western Sydney University, Penrith, NSW Australia; 4 Department of Pharmacy, DIFAR, University of Genova, Viale Benedetto XV, 3, 16132 Genoa, Italy; 5 Department of Neuroscience, Genetics, Maternal and Child Health, DINOGMI, University of Genova, Largo P. Daneo 3, 16132 RehabilitationGenoa, Ophthalmology Italy; 6 Division of Clinical Neurophysiology, Department of Neuroscience, IRCCS Ospedale Policlinico San Martino, Largo R. Benzi 10, 16132 Genoa, Italy; 7 DIBRIS, University of Genova, Via Opera Pia, 13, 16145 Genoa, Italy; 8 Department of Mathematics, Computer Science and Physics, University of Udine, Via delle Scienze, 206, 33100 Udine, Italy

**Keywords:** GABAergic transmission, Loop network, Somatosensory cortex, Systems and Control Theory, Thalamic reticular nucleus, Thalamus, Ventroposterolateral nucleus

## Abstract

Fibromyalgia (FM) is an unsolved central pain processing disturbance. We aim to provide a unifying model for FM pathogenesis based on a loop network involving thalamocortical regions, i.e., the ventroposterior lateral thalamus (VPL), the somatosensory cortex (SC), and the thalamic reticular nucleus (TRN). The dynamics of the loop have been described by three differential equations having neuron mean firing rates as variables and containing Hill functions to model mutual interactions among the loop elements. A computational analysis conducted with MATLAB has shown a transition from monostability to bistability of the loop behavior for a weakening of GABAergic transmission between TRN and VPL. This involves the appearance of a high-firing-rate steady state, which becomes dominant and is assumed to represent pathogenic pain processing giving rise to chronic pain. Our model is consistent with a bulk of literature evidence, such as neuroimaging and pharmacological data collected on FM patients, and with correlations between FM and immunoendocrine conditions, such as stress, perimenopause, chronic inflammation, obesity, and chronic dizziness. The model suggests that critical targets for FM treatment are to be found among immunoendocrine pathways leading to GABA/glutamate imbalance having an impact on the thalamocortical system.

**Supplementary Information:**

The online version contains supplementary material available at 10.1007/s10827-022-00826-8.

## Introduction

Fibromyalgia (FM) can be defined as an idiopathic, chronic pain syndrome characterized by wide-spread, multi-site pain and a complex of accompanying symptoms, including sleep problems, fatigue, perception and mood disorders, anxiety, cognitive dysfunction, headache, and irritable bowel (Bazzichi et al., 2012, Sarzi-Puttini et al., 2021). The syndrome is affecting about 2% of the general population and it is more prevalent in women (3.4%) than men (0.5%), especially women between 30 and 50 years of age (Neumann & Buskila, 2003).

FM belongs to a group of idiopathic pain conditions, collectively known as Chronic Widespread Pain (Fink et al., 2005), a cluster of disorders created to unify diseases that are often difficult to discriminate from each other. Its diagnosis is challenging due to the lack of clear objective biomarkers and symptom overlapping with other disorders, and therefore, diagnostic criteria have been codified and updated several times in the past years by the American College of Rheumatology (ACR) and the American Pain Society (Arnold et al., 2019; Wolfe et al., 2016). FM diagnosis is generally given to individuals with chronic widespread musculoskeletal pain for which no alternative cause, such as tissue inflammation or damage, can be identified (Clauw, 2009, Sarzi-Puttini et al., 2021). Along with difficulties in diagnosis, the management of FM patients is problematic and no resolutive treatment is presently available (Ablin et al., 2013).

As frequently occurs in health sciences, the clinical problems of FM are directly linked to the poor knowledge of its etiology. Several different pathophysiological hypotheses have been postulated, but they are disjoint from one another, and a unifying theory is lacking. By following major symptoms, FM has been first considered a peripheral musculoskeletal disorder. However, thanks to innovations in pain management, as well as experimental pain testing, functional neuroimaging, and genetical tests, FM is now recognized as a central disorder, and there is extensive evidence that abnormalities in central pain processing occur in FM patients (Geel, 1994). Neural pathway alterations are believed to result in nociceptive hypersensitivity, causing hyperalgesia, allodynia, referred pain across multiple spinal segments, and chronic widespread pain, together with hypersensitivity to non-nociceptive visual, hearing, and tactile stimuli (Meeus & Nijs, 2007). Nevertheless, patients are left often with unsuccessful therapies, resulting in poor overall wellbeing and mental health (Jahan et al., 2012).

Current therapeutic options are focusing on reducing pain and improving the quality of sleep and physical function through a reduction in associated symptoms (Bellato et al., 2012). However, more tangible, effective treatments are still unavailable. Focusing on the FM underlying pathogenic mechanism is therefore imperative to further advance the current management and understanding of the disease (Hurtig et al., 2001; Mease, 2005). Several different pathophysiological hypotheses have been postulated, including impairment of the hypothalamic–pituitary–adrenal axis, and alterations in specific neurotransmitters such as substance P, glutamate, norepinephrine and serotonin (Bazzichi et al., 2006). Nevertheless, these hypotheses do not provide a synthetic explanation of the pathophysiological mechanism, whereas a unifying theory about this mechanism should take into account both triggering factors from endocrine and immune system and pain processing networks.

A bulk of epidemiological, clinical, neurophysiological and neuroimaging data about FM are available in the literature. Nevertheless, no general paradigm has been formulated yet, allowing to consistently embody this wide information set within a synthetic, unifying framework able to explain the causes of the disease. We aim to formulate a new model of FM pathophysiology based on a hypothesis of pain processing dysfunction in a thalamocortical loop network, and deriving from a combination of literature data with the notions of Systems and Control Theory (Sontag, 2004).

## Identification of a thalamocortical loop in literature data

Considering that pain is the main symptom of FM, our study is focused on pain processing pathways. Studies concerning central pathogenic mechanisms of FM have considered dorsal horns at the intersection site of ascending and descending pain processing pathways (Staud et al., 2021). However, the partial or insufficient efficacy of pharmacological treatments aimed at enhancing descending pain control, e.g. acting on serotonin (Ossipov et al., 2014), opioid, and cannabinoid receptors (Littlejohn et al., 2016), suggests the need of exploring other possible central sites of FM insurgence.

Physiological pain detection and processing in the nervous system starts from nociceptive C,Aδ, and Aβ primary neurons, whose sensory endings are distributed across peripheral tissues. These fibers innervate spinal cord dorsal horns, where peripheral sensory input and supraspinal descending modulation are integrated. Projection neurons from the dorsal horns transmit processed stimuli through spinothalamic, spinomesencephalic, spinoreticular, spinolimbic, and spinocervical pathways. The thalamus is the major supraspinal relay site for ascending pain stimuli, while neuroimaging studies have shown the involvement of various other brain areas, mostly including primary and secondary somatosensory cortices, insula, anterior cingulate cortex, and prefrontal cortex, collectively known as the Pain Matrix (Iannetti & Mouraux, 2010). Pain-related brain activation also includes posterior parietal cortex, basal ganglia, amygdala, hippocampus, brainstem, and cerebellum (Morton et al., 2016). In addition to ascending pathways, descending analgesia systems are also involved in pain managing, including periaqueductal gray matter, locus coeruleus, parabrachial area, and various components of the limbic system (Willis & Westlund, 1997). Different alterations of connectivity among brain areas have been revealed in chronic pain disorders (Apkarian et al., 2005). Acute pain activates the somatosensory cortex, as well as other cortical regions including insular, cingulate, and prefrontal cortices, whereas chronic and neuropathic pain involves much less brain activity. Therefore, it has been hypothesized that chronic pain is the result of an altered thalamocortical activity that is interpreted as pain perception and awareness (Henderson et al., 2013).

Within the above system of pain processing pathways, a loop network can be identified involving thalamocortical regions, *i.e.* the thalamic ventroposterolateral nucleus (VPL), the somatosensory cortex (SC), and the thalamic reticular nucleus (TRN) (Groh et al., 2018). The network includes excitatory, first order glutamatergic fibers of the VPL projecting to the primary SC and TRN, glutamatergic fibers from the primary SC projecting to the first order neurons of VPL and to TRN, and inhibitory y-aminobutyric acid (GABA) projections from TRN to VPL (Lam & Sherman, 2011). First order thalamic neurons actually target the primary SC layer 4 (L4), while feedback cortical glutamatergic fibers to VPL and TRN come from layer 6 (L6) (Takata, 2020). However, it has been shown that L4 acts as a distributor of intracortical excitation thus making L4 and L6 excitatorily coupled (Feldmeyer, 2012), and therefore, the primary SC can be considered as a single functional element in the loop system.

## Development of a loop model of FM pathogenesis

### Systems and Control Theory background

Feedback loop control allows a system to use its output to modulate its input-dependent activity, while it has been proposed that this kind of mechanisms represent the key to understand life processes (Burlando, 2017; El-Samad, 2021). According to Systems and Control Theory, physiological processes can be related to the presence of feedback loops of different sign. Homeostasis, deputed to maintain the functional *status quo* (pH, temperature, redox balance, osmolarity), is related to negative loops, *i.e.* closed chains having one or, more in general, an odd number of inhibitory steps. Conversely, the switching among different functional states deputed to produce changes (growth, development, differentiation, adaptation, and reproduction) requires multistability (namely, the presence of multiple stable equilibrium points), which depends on the activity of positive loops, *i.e.* closed chains with no inhibitory steps or an even number of them (Blanchini et al., 2014, Blanchini et al., 2015). Bistable transitions, where the two stable steady states correspond to healthy and sick conditions, have been suggested to explain the pathophysiology of numerous diseases, including, among others, cramps and myokymia (Baldissera et al., 1994), prion diseases (Kellershohn & Laurent, 2001), autoimmune diseases (Rapin et al., 2011), Alzheimer’s disease (De Caluwe & Dupont, 2013), and type II diabetes (Wang, 2014).

### Hybrid negative/positive loop network

**Fig. 1:**
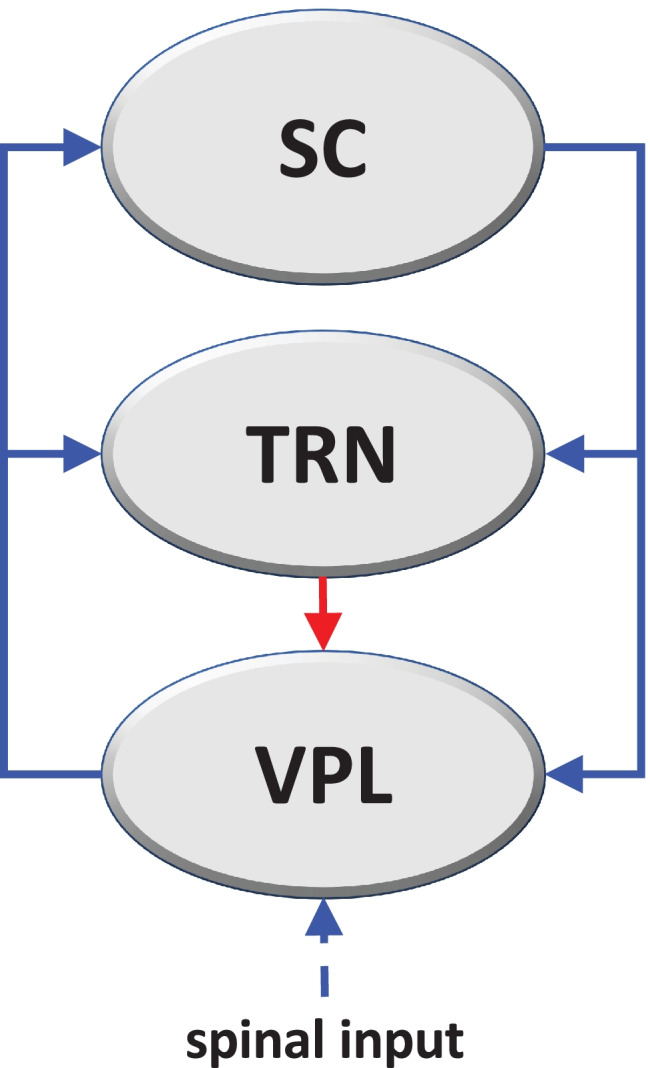
Diagram of the thalamocortical loop system that drives the pathophysiological transition to FM. The system consists of three brain regions, i.e. primary somatosensory cortex (SC), thalamic reticular nucleus (TRN), and thalamic ventroposterolateral nucleus (VPL), connected by excitatory glutamatergic (blue) or inhibitory GABAergic (red) fibers. Three loops can thus be identified, a positive one: VPL-SC-VPL, having excitatory steps only, and two negative ones: VPL-SC-TRN-VPL and VPL-TRN-VPL, both having one inhibitory step. Based on the strength of the inhibitory step with respect to excitatory ones, the system’s global behavior can be dominated by the effect of negative or positive loops

In the above thalamocortical network, three loops can be identified, a positive one: VPL-SC-VPL, having excitatory steps only, and two negative ones: VPL-SC-TRN-VPL and VPL-TRN-VPL, both having one inhibitory step (Fig. 1). Depending on the strength of the various interactions, this system can globally behave as a candidate oscillator if the effect of the negative loop dominates (either having a single equilibrium point that is stable or yielding persistent oscillations), or as a candidate multistationary system if the effect of the positive loop dominates (with multiple stable equilibrium points) (Blanchini et al., 2014).

A downward shift of the GABAergic/glutamatergic strength ratio would tend to abolish the negative loop contribution: the positive loop would then dominate, and thereby induce a multi-stationary behavior. Multi-stationary systems, *i.e.* dynamic systems having at least two stable equilibrium points, are of interest in pathogenesis since they are known to drive transitions in biological systems, also including the insurgence of disease (Doig, 2018). As a result of this coexistence of regimes, the network can be driven from “healthy” to “pathological” by an imbalance in neurotransmitters.

### Mathematical model of the loop network

The loop system visualized in Fig. 1 represents the interactions among different brain areas, and its dynamics are mathematically described by a system of differential equations with three variables, representing mean firing rates (i.e. number of spikes per second). The system of differential equations is reported in detail in the Appendix section, and is schematically represented as follows:

1 $$\tau \dot{S}+S=f\left(V\right)$$

2 $$\tau \dot{T}+T=f\left(V+S\right)$$

3 $$\tau \dot{V}+V=\frac{g\left(aT\right)\cdot {S}^{p}}{{{h\left(aT\right)}^{p}+S}^{p}}$$

where $\dot{x}$ denotes the time derivative of *x,* the variables *S*, *T*, and *V* represent the mean firing rates of neuron populations belonging to SC, TRN, and VPL, respectively, the parameter *a* is a coefficient that varies in the interval 0 ≤ *a* ≤ 1, representing the efficacy of the GABAergic activity, and the functions appearing in the differential equations are assumed to have Hill-type expressions (Ferrante et al., 2009), with *f(x)* being an increasing Hill function, *h(x)* an increasing Hill function plus a constant, and *g(x)* a decreasing Hill function. The variables *S*, *T*, and *V* are real numbers varying between zero and a maximal value (see Supplementary Information). Neuron baseline firing rate has been set to zero.

Replacing the effect of many neurons with a single overall characteristic is a simplification of the considered biological phenomenon. However, from a mathematical standpoint, this simplification does not affect the conclusions in our case: in view of the structure of the problem, the essence of the phenomenon is still captured. The instability we are analyzing, leading to bistability, remains qualitatively unaffected if we consider a “lumped” effect instead of a cascade of individual effects.

Simple models that capture the essential aspects of the phenomenon are useful because they are amenable for analysis and can provide explanations and insight into the functioning of the system.

### Acquisition of bistability for inhibitory GABAergic weakening

**Fig. 2:**
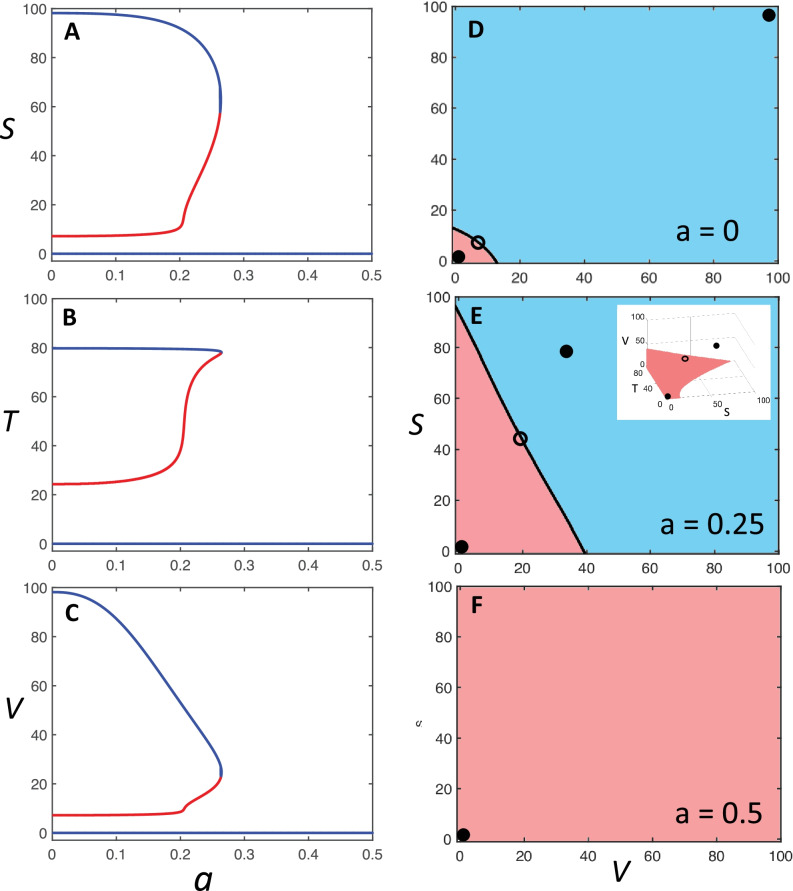
Bifurcation diagrams and phase portraits of the loop system. (**A**, **B**, **C**) Bifurcation diagrams: curves reporting the steady states of variables *S* (panel **A**), *T* (panel **B**), and *V* (panel **C**) as a function of the bifurcation parameter *a*, which represents the strength of inhibitory GABAergic transmission, i.e. the fraction of its full efficiency. The values of *S*, *T*, and *V* are expressed as mean firing rate (Hz), while *a* is a coefficient ranging in the interval 0 – 1, of which only the subset 0 – 0.5 is shown. Curve branches in blue represent stable equilibrium points, or steady states, and branches in red unstable ones. Upon decrease of *a*, the system reaches a bifurcation point where it undergoes a transition from monostability, with a single stable equilibrium point, to bistability, with two stable equilibrium points and an unstable one. The low-firing-rate steady state is assumed to represent the physiological condition, while the high-firing-rate steady state represents the pathogenic condition. (D, E, F) Bidimensional phase portraits in the *V*/*S* plane showing the basins of attraction of stable equilibrium points (black filled dots) for different values of *a* and for *T* = 80, i.e. the maximal value of the *T* variable, at which the basin of attraction of the low-firing-rate equilibrium point is the widest. The basin of attraction of the high-firing-rate steady state is in light blue, and that of the low-firing-rate steady state in light red. Unstable equilibrium points (open dots) are also shown. The plot in panel E corresponds to a value of $a$ immediately below the bifurcation point, while the inset shows the corresponding 3D phase portrait in the *S*/*T*/*V* space, where only the low-firing-rate basin of attraction is colored (in light red).

The analysis of the system dynamics performed in MATLAB (see Supplementary Information) shows that, when the inhibitory GABAergic function is completely active (*a* = 1), the system is monostable with a steady state characterized by low firing rate for all the elements of the loop system (Fig. 2). Under this condition, the system phase portrait has a single basin of attraction (associated with the unique equilibrium point); therefore, regardless of the excitation level at which each element may be induced, the time evolution of the system will follow a trajectory that converges to the low-firing-rate steady state (Fig. 2). However, if the efficacy of the GABAergic fibers scales down, for *a* = 0.265 the system crosses a bifurcation point and becomes bistable, thereby acquiring two distinct, low- and high-firing-rate steady states. In this new scenario, the system phase portrait has two basins of attraction, one for each of the two stable equilibrium points: the system will converge to one of the steady states if the initial conditions lie within the corresponding basin of attraction (Fig. 2). Moreover, the basin of attraction of the high-firing-rate steady state enlarges as *a* decreases, which renders the system more and more prone to converging to the high-firing-rate steady state (Fig. 2).

More specifically, for values of *a* immediately smaller than the bifurcation value, *V* at the high equilibrium point is relatively low (about 40 Hz), whereas *S* is much higher (about 80 Hz) (Fig. 2). Hence, if the value of *a* is slightly smaller than the bifurcation value, a very small activation of the VPL nucleus, e.g. by spinothalamic input, is sufficient to drive the SC cortex to a high activation level that becomes permanent, i.e. the system shows an ultrasensitive behavior, typical of a biological bistable switch (Ferrell & Machleder, 1998).

 It is worth highlighting that the results displayed in our simulations hold qualitatively for a general class of systems including our model (1)-(3), regardless of the specific parameter values. The thorough mathematical analysis we provide in the Supplementary Information (SI) shows that the system always admits the equilibrium at zero (corresponding to baseline activity for all neuron populations), which is always stable, and may overall admit an odd number of equilibria, which are always ordered and are typically three: the “low” stable equilibrium at zero, which corresponds to basal activity, the “high-firing-rate” stable equilibrium, which can be related to a pathogenic pain processing activity, and an unstable equilibrium with intermediate neuron activity (see SI, Sects. 2.2 and 2.3). If such a bistability pattern emerges for $a=0$, we show that it is preserved for values of $a$ below a certain threshold ${a}^{*}$, $0<a<{a}^{*}$ (see SI, Sects. 2.2 and 2.3). We also prove that there exists a threshold value $\widehat{a}$ for $a$ (associated with the bifurcation value observed in our numerical simulations) such that, for all $a>\widehat{a}$, the only admissible equilibrium is the one at zero (associated with basal activity), which is stable and hence has the whole state space as a basin of attraction (see SI, Sect. 2.4). Although the numerical value of the threshold depends of course on the parameters, the qualitative behavior is the same, independent of the chosen parameter values for the system (1)-(3): GABAergic weakening can induce bistability, while strengthening GABAergic activity above a certain threshold level always guarantees that the only possible equilibrium is the baseline-firing-rate steady state.

The qualitative model behavior is also robust with respect to the form of the functions and the values of parameters considered in the differential equations. For instance, if one of the Hill functions appearing in (3) is substituted by a fixed parameter, a bifurcation point is always observed, although shifted to the right at a higher value of *a*. Finally, if the GABAergic function is reversed turning to excitatory (e.g. for an inversion of the chloride gradient across the cell membrane, see below), so Eq. (3) becomes:

4 $$\tau \dot{V}+V=f\left(S+T\right),$$

the system shows bistability in the whole interval 0 ≤ *a* ≤ 1, as expected based on previous structural classification (Blanchini et al., 2014), with a much wider attraction basin of the high-firing rate steady state throughout. We assume that the high-firing-rate steady state represents a pathogenic pain processing activity that gives rise to chronic pain condition. Therefore, the emergence of bistability in the system dynamics is the essential prerequisite for the development of FM through a bistable switch behavior. Coefficient *a* is the bifurcation parameter that upon decrease can turn the system behavior from monostable to bistable. Therefore, in our model a reduction of GABAergic efficacy is the triggering event of the FM pathogenic process. Consequently, under our hypothesis, pharmacological and clinical strategies aimed at a successful FM management should search for suitable targets among biochemical pathways affecting the TRN GABA system.

## Support to the model from literature data

### Immunoendocrine effects on GABAergic transmission

Our model describes the activity of a brain network and embodies the consequences of external modulatory agents on the network dynamics. These modulations are thought to depend on a complex of immunoendocrine activities whose comprehensive effect on the brain network is represented by the coefficient *a*. Representing endocrine and immunological actions in a lumped fashion through coefficient *a* does not affect model predictions, as long as we can assume that *a* is an external input governed by factors that are not in turn affected by the *T*, *S*, and *V* variables.

Several data suggest the involvement of the endocrine system in FM, such as biased gender ratio with female prevalence (Arout et al., 2018), correlation with stress (Fries et al., 2005) and gonadal hormone dysfunctions (Bazzichi et al., 2012). On the other hand, the immune system also interferes with pain processing and a possible role of immune responses in FM pathogenesis has been envisaged (Kadetoff et al., 2012). Increased levels of inflammatory cytokines and chemokines in both plasmatic (Ernberg et al., 2018; Rodriguez-Pinto et al., 2014) and cerebrospinal fluids (CSF) (Kosek et al., 2015) of FM patients have been found. The CSF is a potential “mirror” for pathophysiological dysfunctions of the spinal cord, however, many questions remain to be addressed, such as if this inflammatory fingerprint may be a predisposing factor or a cascade event of FM (Menzies & Lyon, 2010, Backryd et al., 2017). An autoimmune reaction directed to nervous tissue, possibly triggered by infections, has also been invoked as a possible pathogenesis mechanism of FM (Meester et al., 2019). Accordingly, a gene expression survey has revealed the modulation of genes involved in immunological pathways connected to interleukin-17 (IL-17) and to type I interferon signatures, thus strengthening the idea of a role of autoimmunity in the disease (Dolcino et al., 2020).

As stated above, the GABAergic, inhibitory pathway connecting TRN and VPL can be regarded as a crucial element for the acquisition of bistability by our putative pathogenic network. Therefore, we are focusing on immunoendocrine activities that could affect the GABAergic function. Alternatively, an increase of glutamatergic function could also be envisaged.

A first link between GABA and FM is suggested by the negative influence of various factors associated with the disease, such as female sex, obesity, and stress, on glutamic acid decarboxylase (GAD), the rate-limiting enzyme in the conversion of glutamate to GABA (Fitzgerald & Carter, 2011). Accordingly, neuroimaging and pharmacological data collected on FM patients argue for GABA/glutamate imbalance in thalamocortical networks. These include the finding of low TRN activity and reduced GABA levels (Foerster et al., 2012; Henderson et al., 2013), the improvement of pain symptoms due to GABA rise and glutamate decrease obtained by transcranial direct current stimulation (tDCS) (Foerster et al., 2015), and the efficacy of drugs like pregabalin and gabapentin that can increase GABA and reduce glutamate release (Sluka & Clauw, 2016). In addition, certain immunoendocrine stimuli are known to affect central GABA levels. GABA reduction has been observed in perimenopausal and postmenopausal women (Wang et al., 2016, 2019), which correlates with higher FM prevalence in women and its connection with climacteric symptoms (Carranza-Lira & Hernandez, 2014). From this point of view, FM seems to be part of a series of neurological disorders showing perimenopausal prevalence and correlation with gonadal hormone alterations, such as migraine, central vestibular problems, anxiety, and depression (Loder et al., 2007; Mucci et al., 2018; Ozdemir et al., 2020).

Various interrelationships between steroid hormones and the GABAergic function have emerged from different studies. The progesterone-derived neurosteroid, 3α-hydroxy-5α-pregnan-20-one (allopregnanolone), is a positive allosteric modulator of GABA_A_ receptors (GABA_A_R) (Reddy, 2010), providing a possible mechanism for the negative repercussion of menopause on GABAergic function. Moreover, sex- and stress-derived neurosteroids like allopregnanolone and 5α,3α-tetrahydrodeoxycorticosterone (THDOC) are positive modulators of GABA_A_R, but contrasting effects of these steroids, including anxiety, irritability, and aggression have also been found, leading to discover that the effects of neurosteroids on the mood show a biphasic, U-shaped relationships, depending on their concentration (Backstrom et al., 2011; Shen et al., 2007). Such peculiar dose–response has been explained with an inversion in neurosteroid modulation, from activation to inhibition, of GABA_A_R, specifically the α_4_β_2_δ subunit combination, which is most sensitive to neurosteroids (Wohlfarth et al., 2002). The inversion occurs when the Cl^–^ gradient across the cell membrane causes excitatory, *i.e.* depolarizing rather than hyperpolarizing, currents after GABA_A_R activation (Hewitt et al., 2009; Kahle et al., 2008). Hence, stress would induce a Cl^–^ gradient similar to that of fetal and early postnatal life periods (Kahle et al., 2008). In the adult brain, the Cl^–^ gradient is primarily maintained by the KCC2 K^+^/Cl^−^ cotransporter that is positively regulated by phosphorylation at the Ser940 residue. It has been proposed that KCC2 is dephosphorylated during stress conditions, thereby leading to a weakening of the Cl^–^ gradient up to its inversion and a consequent excitatory activity of GABAergic fibers (Mody & Maguire, 2011). It remains to be ascertained whether synaptic (phasic) or non-synaptic (tonic) transmission is mostly affected, since the GABA_A_R δ subunit is prevalently an extra-synaptic component (Hannan et al., 2020). Given that δ-GABA_A_R are also expressed in the thalamus (Belelli et al., 2005), the paradoxical effect of stress-derived neurosteroids could account for the downregulation of GABAergic transmission envisaged by our pathogenesis model, thus providing a mechanistic basis for the correlation between stress and FM.

Evidence is also accumulating that stress impairs glial cell function and glutamate clearance in the hippocampus and prefrontal cortex, leading to glutamate accumulation at the synaptic cleft and consequent excitotoxicity (Popoli et al., 2011). It has been proposed that chronic stress induces mineralcorticoid/glucocorticoid receptor (MR/GR) imbalance, thereby impairing the GABA/glutamate ratio (de Kloet, 2014; Mifsud & Reul, 2018), while acute stress or glucocorticoid (GC) administration enhance glutamate release in the hypothalamus, amygdala, and hippocampus (Musazzi et al., 2010). At the same time, GC binding to post-synaptic membrane MRs results in the inhibition of type A potassium currents and facilitates the membrane diffusion of AMPA receptors. However, compensating effects can also occur, since GCs activate post-synaptic membrane GRs, which induce the release of retrograde endocannabinoids able to activate pre-synaptic cannabinoid receptor type 1, which inhibits glutamate release (Groeneweg et al., 2011; Venero & Borrell, 1999).

Besides steroid hormones, various effects of inflammatory cytokines have been reported on GABA release and GABA_A_R activity. It has been shown that IL-6 (Garcia-Oscos et al., 2012), an inflammatory mediator correlated to stress and cortisol, and IL-17 released by astrocytes (Luo et al. 2019), decrease the ratio between inhibition and excitation in rat temporal cortex. These effects have been related to allodynia and chemotherapy-induced peripheral neuropathy, but a link could be established with FM too, since increased levels of IL-17 have been found in FM patients (Pernambuco et al., 2013). In addition, different studies indicate that IL-1β dampens the inhibitory GABAergic function thereby promoting hyperexcitability associated to pathological conditions (Rizzo et al., 2018; Wang et al., 2000; Yan et al., 2015), but contrasting data have been also reported (Brambilla et al., 2007; Miller & Fahey, 1994; Zhu et al., 2006). Tumor necrosis factor (TNF)-α has been shown to downregulate GABAergic synapses, by inhibiting GABA release (De Laurentiis et al., 2000), reducing cell-surface GABA_A_R (Pribiag & Stellwagen, 2013), and decreasing the ratio between GABA and α-amino-3-hydroxy-5-methyl-4-isoxazolepropionic acid (AMPA) synaptic activities (Olmos & Llado, 2014; Stellwagen et al., 2005). However, divergent results have been reported also for TNF-α modulation of GABA_A_R trafficking to the plasma membrane (Stuck et al., 2012). Finally, inhibition of GABAergic activities has been reported for IL-2 (Rozsa et al., 1997; Sawada et al., 1992) and IL-10 (Suryanarayanan et al., 2016). In summary, data about the effects of inflammatory cytokines on GABAergic functions are variegated and not always consistent, but evidence exists that some immune responses can decrease the ratio between inhibitory and excitatory activities.

Finally, FM insurgence seems to also be affected by calcitonin gene related peptide (CGRP) and brain-derived neurotrophic factor (BDNF). Increased levels of CGRP in the anterior cingulate cortex and insula correspond to increased excitatory transmission of neurons in these areas (Liu et al., 2020). CGRP appears to be related to increased BDNF levels (Buldyrev et al., 2006), and accordingly, an alteration in BDNF levels has been observed in FM patients (Alves et al., 2020, Polli et al., 2020). The BDNF precursor pro-BDNF can affect the GABAergic transmission by interacting with its p75 receptor and leading to a reduction of the KCC2 Cl^–^ transporter at the cell surface, with consequent lowering of the Cl^–^ gradient, up to its inversion (Porcher et al., 2018). Also, a BDNF role in mediating delayed effects of early life stress is suggested by an interplay between GCs and BDNF (Daskalakis et al., 2015). However, stress and GCs are also known to repress BDNF expression (Chen et al., 2017), showing how the role of BDNF in stress-related pathogenesis is still to be clarified.

### FM comorbidity is consistent with the loop model

As stated above, different neuroendocrine and neuroimmune responses are known to affect GABAergic transmission, and to close the circle, FM is correlated with previous or coexistent immunoendocrine conditions, such as altered hypothalamic–pituitary–adrenal stress response, perimenopause endocrine imbalance (Fink et al., 2005), gonadal hormone imbalance (Bazzichi et al., 2012), chronic inflammation (Rodriguez-Pinto et al., 2014, Backryd et al., 2017), and obesity (Ursini et al., 2011). Childhood adversities have also been shown to increase the risk of developing chronic pain, while FM patients often report a history of abuse and being neglected (Coppens et al., 2017). Even prenatal events can act as predisposing factors, since epigenetic mechanisms underlying the programming of stress response and GABA/glutamate balance may be inherited. It has been demonstrated that DNA methylation in the promoter region of the glutamate decarboxylase (GAD) gene decreases GAD expression and thus GABA synthesis, resulting in GABA/glutamate imbalance that can be detrimental for the developing brain (Shaw et al., 2020), thereby possibly predisposing to the pathogenic functional loop identified in our model.

The heterogeneity of FM patients is in line with the different possible triggering stimuli, either endocrine or inflammatory, that according to our model could converge onto the same neural mechanism, thus producing rather uniform symptoms ascribable to FM classification (Kumbhare et al., 2018). By extending this notion, our model provides hints towards the understanding of the neural mechanisms that are at the basis of the wide panel of Chronic Widespread Pain syndromes (Fink et al., 2005).

### Pharmacological approaches to FM are also consistent with the loop model

Clues to the correctness of our hypothesis can be found in the complex of pharmacological treatments that have been clinically tested for FM. These can be schematically divided into drugs acting on ascending pain processing networks, and drugs acting on descending pathways. The former include gabapentinoids and NMDA antagonists, while the latter include antidepressants leading to serotonin and/or noradrenaline increase, or acting on 5-HT receptors, and in addition opioid and cannabinoid analgesics (Tzadok & Ablin, 2020). It is noteworthy that gabapentinoids and NMDA antagonists both act by depressing the glutamatergic function in favor of the GABAergic one, and in addition that pregabalin is one of the three drugs that have been approved in the treatment of FM by the US FDA (Tzadok & Ablin, 2020). This is in line with our model, since these drugs would hinder the transition to bistability of our loop system.

Clinical trials based on self-report measures of pain intensity have reported higher efficacy of antidepressants like duloxetine compared to anticonvulsants like pregabalin (Bidari et al., 2019; Lee & Song, 2016). Given the activity of serotonin–norepinephrine reuptake inhibitor exerted by duloxetine, a dysregulation of descending pain modulation in FM pathogenesis could be hypothesized (Ossipov et al., 2014), but the low efficacy of opioid treatments seems to rule out this possibility (Littlejohn et al., 2016), with the possible exception of the weak opioid tramadol, which however is considered a second-line treatment for severe FM cases only (Tzadok & Ablin, 2020). It can also be argued that duloxetine improves pain perception due to its relationship with the emotional sphere, as supported by the finding that pain catastrophizing is associated with an FM gene polymorphism (Alves et al., 2020).

## Possible validations of the model

We presented a putative unifying model for the pathogenic mechanism of FM, based on improper activation of pain processing. We suggested that a thalamocortical feedback loop system and its GABAergic/glutamatergic strength ratio should be regarded as a crucial hub. Based on literature review, we provided evidence that sustain our hypothesis, focusing on FM-related immunoendocrine imbalance, FM comorbidity, and pharmacological approaches to FM.

The formulated hypothesis could pave the way to a second-level assessments involving patient recruitment. On the experimental ground, to specifically prove a weakening of the GABAergic transmission or a strengthening of the glutamatergic transmission in the VPL and SC regions, Proton Magnetic Resonance Spectroscopy (^1^H-MRS) could be used to measure neurotransmitter levels (Foerster et al., 2015), while the expression of GABA and glutamate receptors could be quantified by Positron-Emission Tomography (PET) with radioligands (Kassenbrock et al., 2016). In addition, variations in connectivity between the VPL and SC regions could be explored by high-resolution Functional Magnetic Resonance Imaging (fMRI) (Cagnie et al., 2014). An accurate survey of the hormonal and inflammatory status, and of the psychological traits and life history of patients (Lee, 2020), could clarify the role of psycho-social and immunoendocrine factors, and their correlation with neurotransmitter imbalance and altered network connectivity.

If all these data fit the herein proposed model, it would deserve the attention of pharmacological and electrotherapy investigations, as a possible basis for the development of new therapeutic treatments and clinical trials.

## Conclusions

We have proposed a thalamocortical loop network as a model of the FM pathogenic mechanism. This network involves first-order, VPL thalamic fibers, the primary SC, and the TRN, and has been chosen because it is the first supraspinal relay of pain stimuli, and can undergo a transition from monostability to bistability for GABAergic weakening. Such a transition is assumed to be the essential step of the pathophysiological process because the bistable system admits both a low-firing-rate steady state, and a high-firing-rate one that can be reached by the bistable system even for weak excitatory spinal inputs to the thalamic VPL nucleus. Hence, the high-firing-rate steady state is assumed to be the pathogenic condition. The model is in line with ideas considering FM as a central problem, and is compatible with the innovative notion of nociplastic pain that has been introduced to define pain processing disorders (Bailly et al., 2020).

Despite indicating a specific thalamocortical brain network, the model leaves open the question about what happens upstream of this system. As we have seen, different immunoendocrine events can have an impact on GABAergic transmission, consistently with the view of FM as a disorder with multifactorial etiology (Ablin et al., 2008). Hence, our model can be considered as a bottleneck towards which different early events are conveyed, varying from patient to patient, or concomitantly/subsequently acting within the same patient. This suggests that modeling immunoendocrine events upstream the identified brain network would be unsuitable to provide a unique model of FM pathogenesis, similarly to what may occur for other multifactorial diseases. Moreover, as explained above, the complex of upstream events acting on the brain network can be mathematically treated as a single parameter in the model, due to the common target of these events in the model and their independence from the model’s variables. Hence, the model represents a unifying step in the disease development, indicating a possible site of therapeutic attack valid for any patient independently of previous history.

## Appendix

We provide here a mathematical model that describes the dynamic evolution of the functional agents involved in the feedback loop system shown in Fig. 1, i.e. the somatosensory cortex (SC), the thalamic reticular nucleus (TRN), and the thalamic ventroposterolateral nucleus (VPL). The activating (blue) and inhibitory (red) interactions visualized in Fig. 1 are modelled in terms of differential equations by using monotonically increasing activation functions, denoted by *f(⋅)*, and a monotonically decreasing inhibition function, denoted by *g(⋅)*. These functions are assumed to have the Hill-type expressions.

5 $$f\left(x\right)=\frac{\alpha {x}^{p}}{{\beta }^{p}+{x}^{p}}$$

6 $$g\left(x\right)=\frac{\gamma }{{\delta }^{p}+{x}^{p}},$$

where *p* is the Hill coefficient and *α, β, γ,* and *δ*, are positive real parameters. The use of Hill functions is justified by a wide set of data showing that they model biological dose–response interactions faithfully, including intracellular signaling networks and neuron firing rates as a function of excitatory or inhibitory stimuli (Huang et al., 2014; Silver, 2010). In addition, it is assumed that each of the above functional agents $x$ is subject to a spontaneous reduction of its activity and evolves with time constant τ; also, $\dot{x}$ denotes the time derivative of *x*.

Then, the dynamics associated with the feedback loop arrangement visualized in Fig. 1 is described by a system of ordinary differential equations. The excitatory activity of the glutamatergic fibers of VPL on SC has been modeled by an increasing Hill function, as frequently reported in input–output responses among neurons (Chabrol et al., 2015; Currin et al., 2020; Ferrante et al., 2009):

7 $$\tau \dot{S}+S={m}_{1}\frac{{V}^{p}}{{e}^{p}+{V}^{p}}$$

where *S* and *V* are the mean firing rates of SC and VPL, respectively, *m*_1_ is maximal output firing rate of glutamatergic fibers, and *e* is the input firing rate that induces half-maximal output firing rate. Other parameters as above.

Similarly, the combined excitatory activity of the glutamatergic fibers of VPL on TRN has been modeled by the following:

8 $$\tau \dot{T}+T={m}_{2}\frac{{(V+S)}^{p}}{{e}^{p}+{(V+S)}^{p}}$$

where *T* is the mean firing rate of TRN, and *m*_2_ is the maximal output firing rate of GABAergic fibers. Other parameters as above.

Finally, the combined excitatory and inhibitory activity exerted by SC and TRN, respectively, on VPL has been modeled by using an increasing Hill function of variable *S*, whose parameters *m* and *e* have been expressed as Hill functions of variable *T*. In different studies, the effects of inhibitory fibers on input–output responses of neurons to excitatory fibers have been reported to occur by either reducing maximal output firing rate, or decreasing the sensitivity of neurons to excitatory stimuli, i.e. a rightward shift of the input–output firing rate curve, or both (Currin et al., 2020; Ferrante et al., 2009). We have therefore considered both effects in the following differential equation, where an increase of *T* induces a decrease of the maximal output firing rate *m*, modeled by a decreasing Hill function, and also induces an increase of the *e* parameter, modeled by an increasing Hill function plus a constant, which represents the basal, unaffected *e*_0_ value. Moreover, to model a reduction of GABAergic efficacy, the variable *T* is multiplied by a coefficient *a* ranging in the interval 0 ≤ *a* ≤ 1. The differential equation is therefore the following:

9 $$\tau \dot{V}+V=\frac{g(aT)\cdot {S}^{p}}{{S}^{p}+{h\left(aT\right)}^{p}}$$

where:

10 $$g(aT)=\frac{{m}_{1}}{1+{\left(\frac{aT}{e}\right)}^{p}}$$

11 $$h(aT)={e}_{0}+{m}_{2} \frac{(a{T)}^{p}}{{e}^{p}+{(aT)}^{p}}$$

If the inhibitory interaction disappears (*a* = 0), then Eq. (9) takes the same form as (7). The computational analysis of the model dynamics has been conducted by using MATLAB (version R2020b, MathWorks, Natick, MS, USA). The values of the parameters of the differential equations are listed in Table 1 and have been chosen based on experimental evidence reported in the literature.

**Table 1:** Model parameters and their nominal values

Symbol	Name	Value	Reference
$\tau$	Time constant measuring the response lag of each variable	0.5 (sec)	(Barardi et al., 2016; Kornijcuk et al., 2016)
${m}_{1}$	Maximal output firing rate of excitatory fibers	100 (Hz)	(Currin et al., 2020; Kanagasabapathi et al., 2012; Olsen et al., 2010)
${m}_{2}$	Maximal output firing rate of inhibitory fibers	80 (Hz)	(Ferrante et al., 2009; Uusisaari et al., 2007)
$e$	Input firing rate inducing half-maximal output	20 (Hz)	(Rothman et al., 2009; Olsen et al., 2010; Li et al. 2017)
$p$	Hill coefficient	2.5	(Rothman et al., 2009; Olsen et al., 2010; Li et al. 2017)
$a$	Coefficient modulating the strength of GABA inhibition	0 – 1	

## Supplementary Information

Below is the link to the electronic supplementary material.

Supplementary file1 (PDF 227 kb)

## Data Availability

Not applicable.

## Code availability

MATLAB codes have been made available online at: http://giuliagiordano.dii.unitn.it/docs/papers/FibromyalgiaCode.zip.
