# Supplementary material for: Thalamocortical bistable switch as a theoretical model of fibromyalgia pathogenesis inferred from a literature survey
Source: J Comput Neurosci. 2022 Jul 11;50(4):471–84. doi: 10.1007/s10827-022-00826-8 (PMC9666334; doi:10.1007/s10827-022-00826-8)
Supplement: Supplementary file 1 — Supplementary file1 (PDF 227 kb) [file 10827_2022_826_MOESM1_ESM.pdf]

Supplementary Information:
Mathematical properties of the model

Thalamocortical bistable switch as a theoretical model of fibromyalgia pathogenesis inferred from a literature survey

Ilaria Demori, Giulia Giordano, Viviana Mucci, Serena Losacco,
Lucio Marinelli, Paolo Massobrio, Franco Blanchini, Bruno Burlando

In this Supplementary Information document, we assess the *structural properties* of the considered model for fibromyalgia pathogenesis (cf. Eqs. (1)-(3) in the main paper) and, in particular, we mathematically analyze the effect of the negative feedback loops, driven by GABAergic activity, on the system behavior.

We consider Eqs. (1)-(3) in the main paper, reported here for ease of perusal,

$$\tau\dot{S} + S = f(V) \tag{1}$$

$$\tau\dot{T} + T = f(V + S) \tag{2}$$

$$\tau\dot{V} + V = \frac{g(aT) \cdot S^p}{h(aT)^p + S^p} \tag{3}$$

where the variables S, T and V represent the mean firing rates of neuron populations belonging to SC, TRN, and VPL, respectively (with the neuron baseline firing rate set to 0), while $f(\cdot)$ is a generic increasing Hill function, $g(\cdot)$ is a generic decreasing Hill function and $h(\cdot)$ is a generic increasing Hill function plus a constant.

The strength of the inhibitory interaction (giving rise to both negative feedback loops) is represented by parameter $a \geq 0$, which represents the efficacy of the inhibitory GABAergic activity.

Here, we prove that the following qualitative properties hold.

- The system **always admits the zero equilibrium** (corresponding to baseline activity for all neuron populations), which is **always stable**.
- When the **inhibitory GABAergic activity** is **absent**, i.e. $a = 0$, the system is **cooperative** and it may exhibit **multistability**. The number of equilibria is then odd (including the zero equilibrium).
- For any possible strength of the GABAergic inhibitory interaction $a \geq 0$, the **equilibria can always be ordered**, namely, at each equilibrium the values of all variables (S, T, V) are larger than the corresponding values at the previous equilibrium. For instance, in the case of three equilibria, the equilibrium values for

the variables S, T, and V are ordered as follows:

$$\begin{aligned}0 &= \bar{S}^0 < \bar{S}^1 < \bar{S}^2 \\ 0 &= \bar{T}^0 < \bar{T}^1 < \bar{T}^2 \\ 0 &= \bar{V}^0 < \bar{V}^1 < \bar{V}^2\end{aligned}$$

- Which type of behavior can we expect for a **small**? If for $a = 0$ there are three equilibria, then the system is **bistable** and it remains bistable for a small enough. In particular, there are two stable equilibria, the "low" zero equilibrium $(\bar{S}^0, \bar{T}^0, \bar{V}^0) = (0, 0, 0)$ and the "high" equilibrium $(\bar{S}^2, \bar{T}^2, \bar{V}^2)$, while the equilibrium $(\bar{S}^1, \bar{T}^1, \bar{V}^1)$ in the middle is unstable. There exists a threshold a^* such that the bistability property remains valid for all $0 \leq a \leq a^*$.
- Which type of behavior can we expect for a **large**? There is a threshold $\bar{a}$ such that, for all $a > \hat{a}$, no positive equilibria can exist: **the only admissible equilibrium is the zero equilibrium** corresponding to baseline neuron activity, $(\bar{S}^0, \bar{T}^0, \bar{V}^0) = (0, 0, 0)$, which is stable.
- Corresponding to the critical value a^*, a single positive equilibrium exists, besides the equilibrium at zero, which is shown to be unstable.

An important conclusion can be drawn based on our model: the transition to a pathological state is not continuous, because a threshold a^* is expected to lead to an abrupt change in the qualitative behaviour. Precisely, a^* corresponds to the transition value above which only the zero (healthy) equilibrium is possible and below which also pathological equilibria exist. For values of a approaching 0, the stable pathological equilibrium becomes the highest possible in terms of the values of $\bar{S}$, $\bar{T}$ and $\bar{V}$.

1 Model Formulation and Assumptions

Definition 1 *A twice differentiable function $f(x)$, defined for $x \geq 0$, is a* sigmoidal function *if $f(0) = 0$ and f is increasing (for positive values of the argument) and asymptotically constant: $\lim_{x\to\infty} f(x) = c$, with $0 < c < \infty$. Moreover, the derivative of f is zero both at 0, $f'(0) = 0$, and at infinity, $\lim_{x\to\infty} f'(x) = 0$, and it has a single maximum for some positive $\hat{x}$.*

An example of a sigmoidal function is the Hill-type expression considered in the main paper:

$$f(x) = \frac{\alpha x^p}{\beta^p + x^p},$$

with $p > 1$ and positive real parameters α and β.

Definition 2 *A twice differentiable function $g(x)$, defined for $x \geq 0$, is a* complementary sigmoidal function *if $g(0) - g(x)$ is a sigmoidal function, namely, if $g(0) = c > 0$ and g is decreasing (for positive values of the argument) and asymptotically zero: $\lim_{x\to\infty} g(x) = 0$. Moreover, the derivative of g is zero both at 0, $g'(0) = 0$ and at infinity $\lim_{x\to\infty} g'(x) = 0$, and it has a single minimum for some positive $\hat{x}$.*

An example of a complementary sigmoidal function is the Hill-type expression considered in the main paper:

$$g(x) = \frac{\gamma}{\delta^p + x^p}$$

with $p > 1$ and positive real parameters γ and δ.

Then, the model considered in the main paper belongs to the general class

$$\tau_S \dot{S} + S = f_1(V) \quad (4)$$

$$\tau_T \dot{T} + T = f_2(V + S) \quad (5)$$

$$\tau_V \dot{V} + V = k(aT, S) \quad (6)$$

where functions f_1 and f_2 are assumed to be sigmoidal (as per Definition 1, see Figure 1 left), while function $k(aT, S)$ is decreasing in the first argument and increasing in the second (see Figure 1 right).

For a fixed value of $T = T_0 > 0$, function $\tilde{k}(S) \doteq k(aT_0, S)$ is sigmoidal (as per Definition 1, hence it has the same properties stated before). Conversely, for a fixed value of $S = S_0 > 0$, $\hat{k}(aT) \doteq k(aT, S_0)$ is a decreasing function of aT, asymptotically converging to 0: $\lim_{aT\to\infty} \hat{k}(aT) = \lim_{aT\to\infty} k(aT, S_0) = 0$. We also assume that

- $k(0, S) > 0$ for all $S > 0$, while $k(0, 0) = 0$
- $k(aT, 0) = 0$ for all $aT \geq 0$
- $\frac{\partial k}{\partial aT} = 0$ for $aT = 0$
- $\frac{\partial k}{\partial S} = 0$ for $S = 0$

An example of such function k is the one considered in the main paper:

$$k(aT, S) = \frac{g(aT) \cdot S^p}{h(aT)^p + S^p},$$

where g is a complementary sigmoidal function (as per Definition 2), and

$$h(aT) = e_0 + m_2 \frac{(aT)^p}{e^p + (aT)^p}.$$

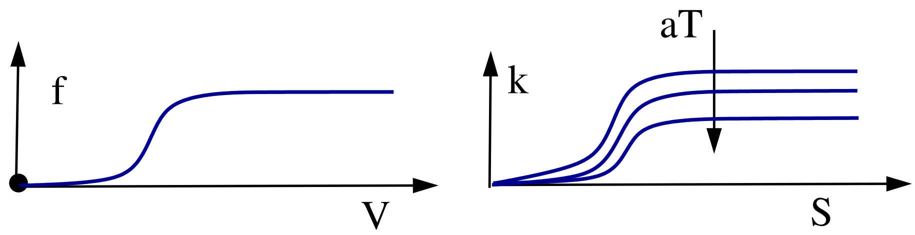

Figure 1: Function $f(V)$ is a sigmoidal function of V (left), while function $k(aT, S)$ is a sigmoidally increasing function of S and a decreasing function of aT (right).

2 Model analysis

We are interested in a parametric investigation with respect to $a \geq 0$ (in the main paper, we focus on $0 \leq a \leq 1$).

2.1 Boundedness

Since the functions f_1, f_2 and k are bounded (they cannot exceed a maximum value, regardless of the values of their arguments), the solutions of the system are bounded due to the presence of the linear terms.

2.2 Equilibria

Let us consider the set of possible equilibria. The zero vector $(\bar{S}^0, \bar{T}^0, \bar{V}^0) = (0,0,0)$ is always an equilibrium, associated with the baseline neuron activity (healthy state). This can be immediately checked by substituting the zero vector in the system equations (4)–(6): then, the resulting derivatives become zero. We wonder whether there are other equilibrium points.

The equilibria are derived by setting $\dot{S} = 0$, $\dot{T} = 0$, $\dot{V} = 0$ and finding the solution of the resulting system of algebraic equations:

$$S = f_1(V) \tag{7}$$
$$T = f_2(V + S) \tag{8}$$
$$V = k(aT, S) \tag{9}$$

As anticipated, $(\bar{S}^0, \bar{T}^0, \bar{V}^0) = (0,0,0)$ is always a solution. In general, from (9) we get

$$\Psi(a, V) = -V + k(af_2(V + f_1(V)), f_1(V)), \tag{10}$$

where we consider V as a variable and a as a parameter. The roots of function Ψ in V correspond to the possible equilibrium values, for a given a.

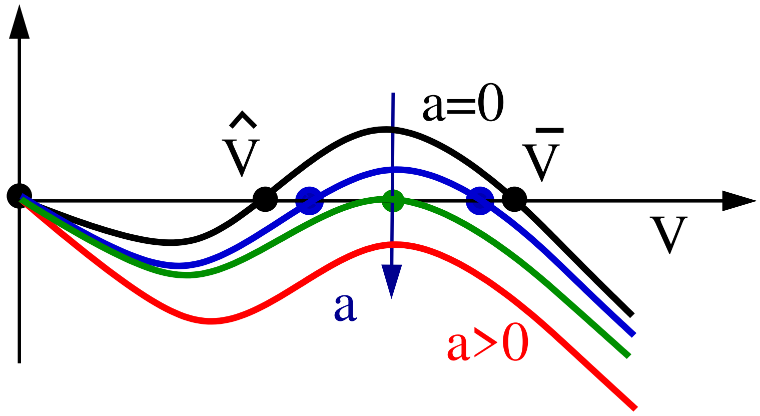

Figure 2: The roots of function $\Psi(a, V)$ in (10) ($\Psi(a, V) = 0$). The value $V = 0$ is always a root, regardless of the value of a. The two positive roots obtained for $a = 0$ (black curve) include all the possible positive roots for $a > 0$ (blue curve) ; in fact, increasing the value of a lowers the curve. The presence of three roots corresponds to the existence of three equilibria for the dynamical system, hence bistability occurs. For $a > 0$ large enough, no positive roots can exist: the only root is the one at zero, hence the only possible equilibrium for the dynamical system is the zero equilibrium associated with baseline neuron activity.

Figure 2 offers a visual intuition of the mathematical analysis.

For fixed $a \geq 0$, the function Ψ as a function of V is initially decreasing and, for large values of V, it converges to $-\infty$, therefore the number of intersections is odd. Consider the curve for $a = 0$ and assume that the largest root of $\Psi(0, V) = 0$ is $\bar{V}$. Then,

since increasing the value of a lowers the curve, for positive values of a all the roots of $\Psi(a, V) = 0$ are smaller than $\bar{V}$.

Before proceeding with our analysis, we introduce the notion of *critical root.*

Definition 3 *Given the equation $f(V) = 0$, a root V^* is* critical *if, besides being a zero of the function, $f(V^*) = 0$, it is a zero also of the function derivative: $f'(V^*) = 0$. For our considered system, we call* critical equilibrium *an equilibrium corresponding to a critical root of function $\Psi(a, V)$.*

We may have a critical root V^* of $\Psi(a, V)$ for some critical value of a, $a = a^*$. In fact, we can have the following possible cases, depicted in Fig. 2.

- For large values of a, there is only the root at zero (cf. the red curve in Fig. 2).
- For small values of a, in addition to the root at zero, there are multiple roots (cf. the blue curve in Fig. 2), all included between the extremal positive roots of $\Psi(0, V)$, corresponding to $a = 0$ (cf. the black curve in Fig. 2, with extremal positive roots $\hat{V}$ and $\bar{V}$).
- There is a critical intermediate value, $a = a^*$, for which a critical root exists (cf. the green curve in Fig. 2), in addition to the root at zero.

We can formalize these findings in the following propositions.

Proposition 1 *Assume that there are no critical roots. Then, the number of nonnegative roots of equation $\Psi(a, V) = 0$ is odd.*

Proof: Let $a \geq 0$. The derivative of function Ψ computed at $V = 0$ is

$$\left.\frac{d\Psi(a, V)}{dV}\right|_{V=0} = -1 + a\frac{\partial k}{\partial(af_2)} f_2'(V + f_1(V))(1 + f_1'(V)) + \left.\frac{\partial k}{\partial f_1} f_1'(V)\right|_{V=0} = -1,$$

because both $f_1'(0) = 0$ and $f_2'(0) = 0$. Hence, for any fixed a, function $\Psi(a, V)$ is negative in a right neighborhood of 0. For V large, function $\Psi(a, V)$ tends to $-\infty$, because function k is a composition of bounded functions, while function $(-V)$ tends to $-\infty$. Therefore, the curve can intersect the abscissa axis an odd number of times overall (counting also the intersection at 0). □

If, for some value $a = a^*$, there is a critical root $V = V^*$ (see the green curve shown in Fig. 2), Proposition 1 still holds provided that the roots are "counted with their multiplicity". Since $\Psi(a^*, V^*) = 0$, $\frac{d}{dV}\Psi(a^*, V^*) = 0$ and the second derivative is non-zero, the Taylor expansion is:

$$\Psi(a^*, V) = \frac{1}{2!}\,\frac{d^2}{dV^2}\Psi(a^*, V^*)(V - V^*)^2 + \frac{1}{3!}\,\frac{d^3}{dV^3}\Psi(a^*, V^*)(V - V^*)^3 + \ldots,$$

and the critical root has multiplicity 2; the roots have a multiplicity corresponding to the index of the first non-zero derivative. Later on, we will analyse the stability properties of the critical equilibrium, corresponding to a critical root, and conclude its instability.

Proposition 2 *Consider the largest (positive) root $\bar{V}$ of $\psi(0, V) = 0$, with $a = 0$, and the corresponding equilibrium values $\bar{S} = f_1(\bar{V})$ and $\bar{T} = f_2(\bar{V} + \bar{S})$. Let $\hat{V}$ be the smallest* positive *root of $\psi(0, V) = 0$, with $a = 0$, and $\hat{S} = f_1(\hat{V})$, $\hat{T} = f_2(\hat{V} + \hat{S})$ the corresponding equilibrium values. Then, for all $a > 0$, all possible equilibria $(\bar{S}_a, \bar{T}_a, \bar{V}_a)$ satisfy*

$$\hat{S} < \bar{S}_a < \bar{S}, \quad \hat{T} < \bar{T}_a < \bar{T}, \quad \hat{V} < \bar{V}_a < \bar{V}.$$

In case no positive equilibria exists for $a = 0$, namely $\bar{V} = \bar{T} = \bar{S} = 0$ is the unique equilibrium when $a = 0$, then no positive equilibrium can exist also for $a > 0$.

Proof: The result follows immediately from the fact that, being function k decreasing in the first argument, increasing the value of a shifts the curve down-word. Therefore, since $\Psi(a, V) \leq \Psi(0, V)$ and $\Psi(0, V) < 0$ for $V > \bar{V}$, the roots $\bar{V}_a$ of $\Psi(a, V) = 0$ are smaller than $\bar{V}$: $\bar{V}_a < \bar{V}$. In turn, $\bar{S}_a = f_1(\bar{V}_a) < f_1(\bar{V}) = \bar{S}$ and $\bar{T}_a = f_2(\bar{V}_a + \bar{S}_a) < f_2(\bar{V} + \bar{S}) = \bar{T}$, because both f_1 and f_2 are increasing functions. The proof for the lower bounds is identical. □

2.3 Stability analysis

We consider the following definition of stability.

Definition 4 *An equilibrium is* stable *if the linearized system around that equilibrium has eigenvalues with strictly negative real part. Otherwise, the equilibrium is* unstable.

In principle, this definition is restrictive, since there are examples of systems whose linearization has eigenvalues with non-positive real part, including eigenvalues with zero real part, and whose trajectories converge to the equilibrium if the initial condition is close enough. However, we do not contemplate this fragile situation as a stable equilibrium, hence we rule out eigenvalues with zero real part, because infinitesimal perturbations can make their real part positive.

We now prove that the healthy equilibrium at zero, corresponding to baseline activity for all neuron populations, is always stable.

Proposition 3 *The equilibrium* $(\bar{S}^0, \bar{T}^0, \bar{V}^0) = (0, 0, 0)$ *is always stable.*

Proof: Since the derivatives of functions f_1, f_2 and k are zero when computed at the origin, the linearized system at the origin is associated with a diagonal state matrix

$$A = \mathrm{diag}\{\ -1/\tau_S,\ \ -1/\tau_T,\ \ -1/\tau_V\},$$

whose eigenvalues are the diagonal elements, which are real and negative. Hence, the equilibrium is stable. □

The theory that follows allows *for any (odd) number of equilibria*. However, to avoid cumbersome notations, we assume that *the equilibria can be at most three*: the possible roots of $\psi(a, V)$, for a given a, are

$$0 = \bar{V}_a^0 < \bar{V}_a^1 < \bar{V}_a^2$$

of which only the zero root surely exists. Correspondingly, the values for S and T are

$$0 = \bar{S}_a^0 < \bar{S}_a^1 = f_1(\bar{V}_a^1) < \bar{S}_a^2 = f_1(\bar{V}_a^2)$$

and

$$0 = \bar{T}_a^0 < \bar{T}_a^1 = f_2(\bar{V}_a^1 + \bar{S}_a^1) < \bar{T}_a^2 = f_2(\bar{V}_a^2 + \bar{S}_a^2)$$

This proves the ordering among the equilibria.

We can also prove the following result.

Proposition 4 *For* $a = 0$*, if there are three ordered equilibria, the zero equilibrium* $(\bar{S}^0, \bar{T}^0, \bar{V}^0) = (0, 0, 0)$ *and the "high" equilibrium* $(\bar{S}^2, \bar{T}^2, \bar{V}^2)$ *are stable, while the medium equilibrium* $(\bar{S}^1, \bar{T}^1, \bar{V}^1)$ *is unstable. There exists a threshold* $a^* > 0$ *such that this pattern is preserved for all* $a < a^*$.

Proof: The result immediately follows from the fact that, for $a = 0$, the system is monotone (see [1] and [2]) and therefore enjoys special properties: its equilibria are ordered and alternating, stable and unstable. The last claim follows by continuity. □

2.4 Equilibria for large a

Finally, the next proposition states that, for a large enough, there are no positive roots for $\Psi(a, V) = 0$.

Proposition 5 *There exists a threshold $\hat{a}$ such that, for $a > \hat{a}$, the only possible equilibrium is the one at zero.*

Proof: Assume that two positive roots exists for $\Psi(0, V) = 0$, denoted as $\hat{V}_0$ and $\bar{V}_0$, with $0 < \hat{V}_0 < \bar{V}_0$. For any a, all other positive roots must be in the interval $[\hat{V}_0, \bar{V}_0]$ (whose extrema are the black dots shown in Figure 2), according to Proposition 2.

Now, by contradiction, assume that no such threshold $\hat{a}$ exists. Hence there exists a sequence $a_j \to \infty$ for which a positive root $\bar{V}_{a_j}$ exists, which must necessarily be within the interval $[\hat{V}_0, \bar{V}_0]$. The sequence of such roots $\{\bar{V}_{a_j}\}$ has an accumulation point $\bar{V}^*$. Without loss of generality, we can assume that $\{\bar{V}_{a_j}\}$ converge to $\bar{V}^*$ (if not, we just need to consider a proper converging sub-sequence). Then, since $\Psi(a_j, V_{a_j}) = 0$ and $\bar{V}_{a_j} \to \bar{V}^*$, necessarily $\Psi(a_j, V^*) \to 0$ for some j. On the other hand, by assumption $k(a_j T, S)$ converges to 0 as $a_j \to \infty$. Since

$$\underbrace{\Psi(a_j, V^*)}_{\to 0} = -V^* + \underbrace{k(a_j f_2(V^* + f_1(V^*)), f_1(V^*))}_{\to 0},$$

then V^* must be 0, which is not possible because $V^* \geq \hat{V}_0 > 0$. The result is therefore proven by contradiction. □

2.5 Critical equilibria

When a critical equilibrium is present, then we can show that it is unstable.

Proposition 6 *Assume that, besides the equilibrium at zero, there exists a critical equilibrium. Then, the critical equilibrium is unstable.*

Proof: Let us rewrite the derivative of $\Psi(a, V)$ as follows:

$$\left.\frac{d\Psi(a,V)}{dV}\right|_{V=V^*} = -1 + a\frac{\partial k}{\partial(aT)} f_2'(V+S)(1 + f_1'(V)) + \left.\frac{\partial k}{\partial S} f_1'(V)\right|_{V=V^*} = 0.$$

The derivative is zero by assumption, because we are considering a critical equilibrium with $S^* = f_1(V^*)$ and $T^* = f_2(V^* + S^*)$. Now consider the Jacobian J of the system factorized as follows:

$$J = \begin{bmatrix} \frac{1}{\tau_S} & 0 & 0 \\ 0 & \frac{1}{\tau_T} & 0 \\ 0 & 0 & \frac{1}{\tau_V} \end{bmatrix} \begin{bmatrix} -1 & 0 & f_1'(V^*) \\ f_2'(V^* + S^*) & -1 & f_2'(V^* + S^*) \\ \frac{\partial k(aT^*, S^*)}{\partial S} & a\frac{\partial k(aT^*, S^*)}{\partial(aT)} & -1 \end{bmatrix}$$

Computing its determinant, $\det(J)$, we immediately check that it is proportional to the derivative $\frac{d\Psi(a,V)}{dV}$, therefore it is zero when computed at $V = V^*$:

$$\det(J) = \frac{1}{\tau_S \tau_T \tau_V} \left.\frac{d\Psi(a,V)}{dV}\right|_{V=V^*} = 0,$$

which is in line with the definition of critical equilibrium. This means that the Jacobian is singular, hence it admits $\lambda = 0$ as an eigenvalue, which implies the instability of the equilibrium, according to our definition. □

Although the scenario with a critical equilibrium is unlikely to occur, because it happens for a specific value of a^*, the analysis is interesting also to understand the case in which two equilibria are very close: in this case we could have, in principle, stability of the upper equilibrium, but its domain of attraction would be very small. This implies that, for the upper (pathological) equilibrium to be stable with a large domain of attraction, the value of a must be sufficiently small.

References

[1] Hirsch, M. W., & Smith, H. (2006). Monotone dynamical systems, In: *Handbook of Differential Equations: Ordinary Differential Equations*, p. 239–357.

[2] Sontag, E. D. (2007). Monotone and near-monotone biochemical networks. *Systems and Synthetic Biology*, 1, 59–87.
